# Effect of Anion-Conducting Electrolytes in Pore-Filling Membranes on Performance and Durability in Water Electrolysis

**DOI:** 10.3390/membranes14120265

**Published:** 2024-12-09

**Authors:** Dahye Jeong, Jin-Soo Park

**Affiliations:** 1 Department of Green Chemical Engineering, College of Engineering, Sangmyung University, Cheonan 31066, Republic of Korea; 2 Future Environment and Energy Research Institute, Sangmyung University, Cheonan 31066, Republic of Korea

**Keywords:** pore-filling membrane, anion exchange membrane, anion-conducting electrolyte, water electrolysis, hydrogen

## Abstract

This study examines the effect of the structural characteristics of anion-conducting monomers within pore-filling anion exchange membranes on the performance and durability of anion exchange membrane water electrolysis. Analysis reveals that acrylamide- and acrylate-based membranes show optimal performance without methyl groups, with acrylamide-based membranes outperforming their acrylate counterparts in current density, particularly at 1.8 V. The AC-AA and AC-MAA monomers demonstrate durability, with AC-MAA showing enhanced alkaline stability, likely due to the presence of a methyl group, resulting in an increase rate of 746.6 μV/h compared to AC-AA’s 1150 μV/h. This study also shows that a commercial membrane exhibits a decrease rate of 3116 μV/h, underscoring the pore-filling membrane’s superior durability. Furthermore, the findings highlight that pore-filling membrane technology enables better durability and performance in electrolysis environments compared to the commercial homogeneous membrane, particularly when alkaline conditions are present. This research provides a foundation for designing high-performance, durable membranes for efficient hydrogen production, particularly under water electrolysis conditions.

## 1. Introduction

Hydrogen is a promising and sustainable energy carrier, contributing to carbon neutrality and supporting a shift towards a low-carbon economy [1,2,3,4,5,6,7,8]. To lessen fossil fuel dependence and curb pollution, renewable-powered water electrolysis is increasingly recognized as an effective method for hydrogen production. Among the available technologies, alkaline water electrolysis (AWE), proton exchange membrane water electrolysis (PEMWE), and anion exchange membrane water electrolysis (AEMWE) are widely employed in both research and industry [9,10,11]. AEMWE, integrating features of both AWE and PEMWE, uses an anion exchange membrane, enabling it to leverage cost-effective catalysts akin to AWE while achieving high-purity hydrogen output comparable to PEMWE. Significant advancements in materials design, component optimization, and system integration are necessary to enhance performance and longevity in hydrogen production systems [12].

Anion exchange membranes (AEMs) are critical components in technologies that require efficient hydroxide ion (OH^−^) conduction. These membranes are made from anion-conducting electrolytes (ACPs), which contain positively charged cationic head groups that facilitate the movement of OH^−^ ions. For optimal performance, AEMs must possess both high ionic conductivity and robust mechanical strength. Various strategies have been developed to enhance these properties such as cross-linking, microphase separation, and composite. First, cross-linking involves creating strong connections between the anion-conducting polymer chains, which improves both the mechanical stability of the membrane and its resistance to alkaline environments. This process helps the membrane maintain its structural integrity under operating conditions while ensuring high ion conductivity [13,14,15,16,17,18,19,20]. Second, microphase separation creates a structure within the membrane that separates hydrophilic (water-attracting) and hydrophobic (water-repelling) regions. The polymer side chains and backbone are organized into a dual-phase configuration that allows for efficient ionic transport. This arrangement promotes evenly distributed ionic domains throughout the membrane, enhancing both ion conductivity and mechanical stability [21,22,23]. Third, in composite membranes, inorganic nanoparticles are embedded into the ACPs, or the membranes are reinforced with materials such as porous or woven substrates. These modifications increase the ion conductivity of the membrane while reducing swelling, making the membrane more durable and suitable for various applications [24,25,26,27,28,29,30].

Emerging approaches aim to boost mechanical and chemical durability and elevate operational temperatures. Pore-filling membranes, created by impregnating porous substrates with polymeric or monomeric electrolytes, offer potential improvements. These substrates are typically hydrophobic polyolefins with high porosity and a small pore size. In pore-filling membranes, ion movement occurs primarily through the cured, pore-filled ACP. Its chemical structure directly impacts ionic conductivity. Hence, selecting compatible combinations of electrolyte monomers and cross-linkers is essential [31,32,33].

This study examines anion exchange pore-filling membranes (AEPFMs), focusing on how polymer hydrophobicity affects membrane properties. Two electrolyte types—one with a methyl group and one without—were combined with a common cross-linker through ultraviolet (UV) curing. It has been reported that methylated monomers generated a more hydrophobic polymer with a higher elastic modulus [34,35,36]. This hydrophobicity difference between the substrate and the filled anion-conducting polymer (ACP) may influence both the performance and durability of water electrolysis.

## 2. Materials and Methods

### 2.1. Substrate Hydrophilization

A porous polyethylene (PE) substrate with a 70 nm pore size, 40% porosity, and 25 µm thickness was used. To enhance the hydrophilicity of the hydrophobic substrate, sodium dodecylbenzenesulfonate (Sigma-Aldrich, Darmstadt, Germany), an anionic surfactant, was applied during pretreatment. Due to the naturally hydrophobic nature of the porous substrate, a hydrophilization process was necessary to create a hydrophilic surface. For this treatment, a 0.5 wt.% surfactant solution in distilled water was prepared. The substrate was washed with ethanol, completely dried at room temperature, and then thoroughly immersed in the surfactant solution for hydrophilization. Finally, it was dried again at room temperature to complete the process.

### 2.2. Preparation of Pore-Filling Membranes

To produce an AEPFM, a photopolymerizable solution was prepared using a homogeneous mixture of anionic conductive monomer electrolytes and a cross-linking agent. The monomers used to produce the AEPFM included photopolymerizable, quaternary ammonium-functionalized vinyl anion-conducting monomers, such as (3-acryloylaminopropyl) trimethylammonium chloride (75 wt.% in water, Sigma-Aldrich, Darmstadt, Germany), [3-(methacryloylamino)propyl] trimethylammonium chloride (50 wt.% in water, Sigma-Aldrich), [2-(acryloyloxy)ethyl]trimethylammonium chloride solution (80 wt.% in water, Sigma-Aldrich), and [2-(methacryloyloxy)ethyl]trimethylammonium chloride solution (75 wt.% in water, Sigma-Aldrich), a cross-linking monomer (1,4-bis(acryloyl)piperazine, >99.0%, Sigma-Aldrich), and a photo-initiator (2-hydroxy-2-methylpropiophenone, 97%, Sigma-Aldrich). Table 1 presents the chemical structures and names of these selected monomers. To form a film on the pretreated substrate, the monomer solution was mixed with anion-conductive and cross-linking monomers and a photo-initiator that was optimized in ratios from 2:1 to 32:1 to determine the best cross-linking degree. The cross-linking monomer solution was prepared at 20 wt.% in distilled water, and 0.5 wt.% photo-initiator (diluted to 10 wt.% in ethanol) was added. The porous substrate was then thoroughly impregnated with the monomer solution to completely fill the pores.

**Figure 1:**
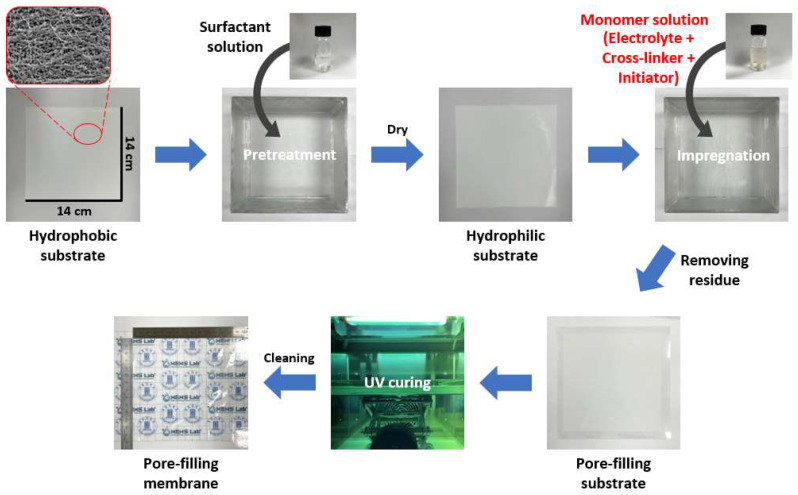
Schematic diagram for the preparation of pore-filling membranes.

The substrate containing the monomer solution was positioned between polyethylene terephthalate (PET) films and pressed with a scraper to remove excess solution from the surface and ensure oxygen exclusion during polymerization. Photopolymerization was performed for 15 min using a 1.5 kW device. Finally, the manufactured AEPFM was polished by washing it with distilled water and drying it at room temperature. Figure 1 illustrates the AEPFM manufacturing process.

### 2.3. Characterization of Membranes

Fourier transform infrared (FT-IR) is an analytical instrument that modifies the wavelength of light within the infrared spectrum to measure the absorption intensity of energy corresponding to the unique vibrational and rotational movements of a specific substance when exposed to light. By analyzing the absorption levels at various wavenumbers, FT-IR can identify a substance’s chemical structure and provide details on the functional groups or molecular motion present. In this study, a FT-IR spectrometer (Spectrum 100, PerkinElmer, Waltham, MA, USA) was utilized to confirm the combination of anionic conductive monomers with cross-linking monomers as well as to characterize the overall chemical structure of the AEPFM. Measurements were conducted within a wavenumber range of 500–4000 cm^−1^.

To determine the contact angle of the AEPFM, a membrane was cut to dimensions of 1 cm × 2 cm and immersed in a 1 M KOH solution for over 24 h to fully exchange the functional groups with OH^−^ ions. After soaking, residual KOH was carefully wiped from the membrane surface, which was then thoroughly air-dried at room temperature. A 3 μL droplet of distilled water was placed on the dried membrane as a sessile drop, and the contact angle was measured using a contact angle measuring device (TL 101, Theta Lite Optical, Biolin Scientific, Stockholm, Sweden) after a 10 s interval.

To measure the areal resistance of the AEPFM, a 2 cm × 2 cm membrane sample was prepared and immersed in a 1 M KOH solution for over 24 h to fully exchange the functional groups with OH^−^ and achieve equilibrium with the KOH solution. The membrane impedance was measured using a potentiostat/galvanostat (SP-150, BioLogics, Seyssinet-Pariset, France) in a clip cell setup at room temperature in the 1 M KOH solution. Impedance measurements were taken at frequencies ranging from 1 MHz to 1 Hz, with an applied amplitude of 20 mV. The membrane’s area resistance (*R_m_*) and ionic conductivity (*σ*) were calculated using Equations (1) and (2), below:

*R_m_* (Ω·cm^2^) = (*R_s_* – *R_b_*) × *A* (1)

(2) $$\sigma \;(S/\mathrm{cm})=\frac{L}{R_{m}}=\frac{L}{{(R}_{s}-R_{b})\cdot A}$$

where *R_m_* (Ω·cm^2^) is the areal resistance of the samples, *R_s_* (Ω) is the impedance of the samples and the background, *R_b_* (Ω) is the impedance of the background (1 M KOH), *A* is the effective area of the samples (cm^2^), *σ* (S/cm) is the ionic conductivity, and *L* (cm) is the membrane thickness.

To evaluate the mechanical properties of the AEPFM, membranes cut to 3 cm × 9 cm were prepared. The dried membranes’ properties were assessed using a universal testing machine (ST-1000, Salt, Incheon, Republic of Korea) following the ASTM D882 method.

To measure the ion exchange capacity of the AEPFM, 2 cm × 2 cm membranes were prepared and placed in a 1 M KOH solution for 24 h, replacing the functional groups with OH^−^ ions. The membranes were then rinsed with distilled water to remove residual KOH and immersed in a 0.01 N HCl solution for 24 h to substitute OH^−^ with Cl^−^. The membrane was then dried at 60 °C for 24 h, and its weight was measured. The 0.01 N HCl solution was titrated with 0.01 M NaOH using an automatic titrator (848 Titrino Plus, Metrohm, Herisau, Switzerland). The ion exchange capacity (*IEC_AEPFM_*) was calculated using Equation (3), below:

(3) $${\mathrm{IEC}}_{\mathrm{AEPFM}}\;(\mathrm{meq}/g)=\frac{C_{NaOH}\cdot (V_{blank}-V_{memb})}{W_{dry}}$$

where *C_NaOH_* (M) is the NaOH molar concentration, *V_blank_* (mL) is the NaOH volume consumed for the titration of the blank, and *V_memb_* (mL) is the NaOH volume consumed for the titration of the membrane.

To measure hydrogen crossover in the AEPFM, a 6 cm × 6 cm membrane sample was prepared and immersed in a 1 M KOH solution for over 24 h to replace the functional groups with OH^−^ ions. A measuring water electrolyzer single cell with the anode and cathode electrodes consisting of a gas diffusion layer (JNT20-A3, JNTG, Hwaseong, Republic of Korea) and a Pt/C catalyst layer (TEC10F50E, TANAKA, Tokyo, Japan) with a catalyst loading of 0.4 mg/cm^2^ was used. A single cell with an active area of 9 cm^2^ was assembled under uniform pressure. It was tested using a water electrolysis station and a potentiostat/galvanostat (SP-150, BioLogics, Seyssinet-Pariset, France) at a differential pressure of 2.5 bar, a cell temperature of 80 °C, and a constant flow rate of hydrogen and nitrogen at 0.2 L/min. With hydrogen supplied to the anode and nitrogen to the cathode, the current due to hydrogen crossover was measured as the voltage was increased at a steady rate [37]. The hydrogen permeability coefficient (k_H_2__) was calculated using Equation (4) [38]:

(4) $$K_{H_{2}}\;(\mathrm{mol}/\mathrm{cm}\cdot s\cdot \mathrm{bar})=\frac{I_{L}\cdot L}{A\cdot n\cdot F\cdot P}$$

where *I_L_* (A) is the limiting current, *L* (cm) is the membrane thickness, *A* (cm^2^) is the active area of the single cell, *n* is the number of electrons in the hydrogen oxidation reaction, *F* is the Faraday constant (96,485 C/mol), and *P* (bar) is the partial pressure.

To assess the alkaline stability of the AEPFM, membranes were prepared by cutting them into 2 cm × 2 cm pieces. These membranes were then placed in a 1 M KOH solution at room temperature for 24 h to ensure that all the functional groups were fully substituted with OH^−^ ions, allowing equilibrium with the 1 M KOH solution. Following this, the membranes were removed from the solution, excess surface solution was wiped off, and they were immersed in a 4 M KOH solution at 60 °C for a period ranging from 0 to 500 h. The 4 M KOH solution was refreshed every 7 days. At each designated time point, the membranes were removed, and membrane impedance was measured in a 1 M KOH solution at 60 °C to calculate the ionic conductivity using Equation (2).

For measuring the electrolysis performance of the AEPFM, membranes were prepared in 6 cm × 6 cm sections. The anode electrode was constructed with a porous transport layer (2GDL09N-025, BEKAERT, Zwevegem, Belgium) and a catalyst layer of IrO_2_ (43396, Alfa Aesar, Haverhill, MA, USA) with a loading of 1.0 mg/cm^2^. The cathode electrode consisted of a gas diffusion layer (JNT20-A3, JNTG, Hwaseong, Republic of Korea) and a catalyst layer of Pt/C (TEC10F50E, TANAKA, Tokyo, Japan) with a loading of 0.4 mg/cm^2^. Both the prepared membranes and electrodes were immersed in a 1 M KOH solution for over 24 h to establish equilibrium with the 1 M KOH solution and to replace all the membrane functional groups with OH^−^ ions. Detailed evaluation conditions are provided in Table 2.

## 3. Results and Discussion

**Figure 2:**
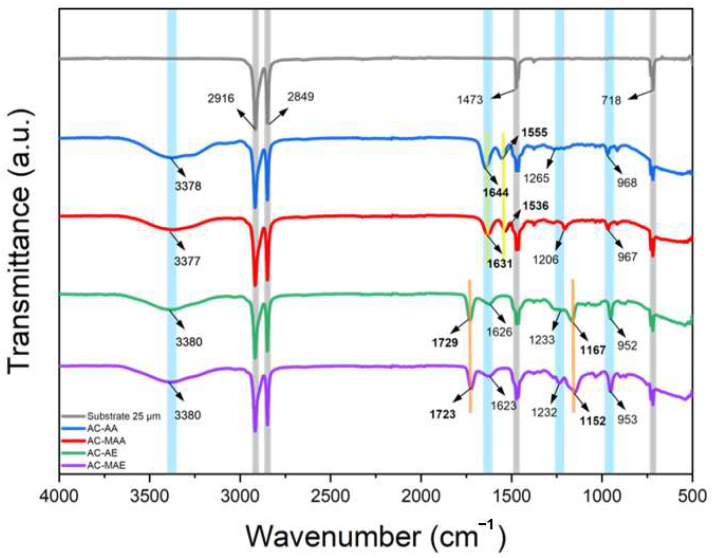
FT-IR Spectra of four different AEPFMs (grey, blue, red, green, and purple lines indicate PE substrate, AC-AA, AC-MAA, AC-AE, and AC-MAE membranes).

In this study, the FT-IR spectra of PE used as a substrate and the prepared AEPFM are shown in Figure 2. PE is a polymer formed by the polymerization of ethylene (C_2_H_4_) units with varying chain lengths (n values). In the PE substrate, four characteristic PE peaks—C-H asymmetrical stretching, C-H symmetrical stretching, CH_2_ scissoring, and CH_2_ rocking vibrations—were observed at 2916, 2849, 1473, and 718 cm^−1^, respectively [39]. As these peaks appeared consistently across all the AEPFM samples, it was concluded that the photopolymerized polymer effectively filled the pores of the PE substrate. In all the AFPFM samples, the N-H stretching and C=O stretching vibrations (Amide I) of the cross-linked monomers appeared in the ranges of 3377−3380 cm^−^¹ and 1623–1644 cm^−1^, respectively. Additionally, N-CH₃ bending and C-N stretching vibrations of the anion-conducting functional groups were observed between 1206–1265 cm^−1^ and 953–968 cm^−1^. For AC-AA and AC-MAA, the -C=O stretching vibration (Amide I) appeared at 1643 and 1631 cm^−1^, respectively, while the N-H bending and C-N stretching vibrations (Amide II) were observed at 1555 and 1536 cm^−1^. In AC-AE and AC-MAE, the –C=O stretching vibration (ester) was seen at 1729 and 1722 cm^−1^, and the –C-O-C symmetric stretching vibration was seen at 1167 and 1152 cm^−1^ [40,41]. These results confirm that the anion-conducting and cross-linking monomers were successfully polymerized, forming the AEPFM.

**Figure 3:**
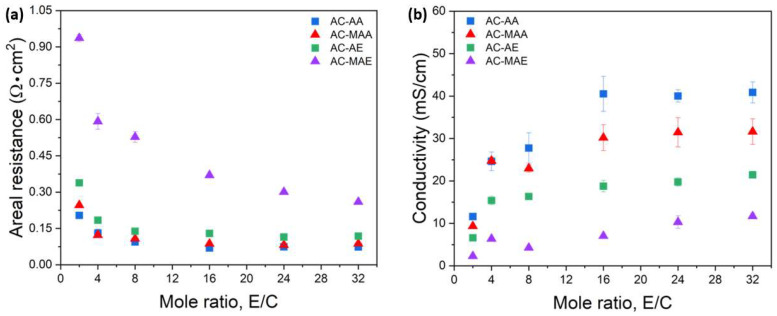
Variation in the (**a**) areal resistance and (**b**) OH^−^ conductivity of AEPFMs as a function of the molar ratios of electrolyte monomer to cross-linker.

The results for the membrane area resistance and ionic conductivity of the AEPFM, manufactured by varying the molar ratios of the selected electrolyte monomer and cross-linking monomer to 2:1, 4:1, 8:1, 16:1, 24:1, and 32:1, are shown in Figure 3. The membrane area resistance decreased significantly as the molar ratio increased from 2:1 to 4:1. After this point, the resistance continued to decrease gradually with increasing ratios from 4:1 to 16:1, with no notable change observed when the ratio was increased further to 32:1. This trend was mirrored in the ionic conductivity, which also rose rapidly as the molar ratio increased from 2:1 to 4:1, then gradually increased from 4:1 to 16:1, with no significant difference observed at ratios beyond 16:1. This outcome suggests that the photopolymerization reaction between the anion-conducting and cross-linking monomers reaches its maximum efficiency at a 16:1 ratio. The degree of cross-linking in the AEM varies with the molar ratio of these monomers: increasing the cross-linking monomer ratio raises cross-linking density, enhancing mechanical strength but reducing molecular mobility, thereby lowering ionic conductivity. Conversely, a higher anion-conducting monomer ratio reduces cross-linking density, increasing molecular mobility and ionic conductivity but decreasing mechanical strength. In this experiment, the PE substrate improved mechanical strength, making the 16:1 ratio ideal for achieving high conductivity. Thus, 16:1 was selected as the optimal molar ratio for further experiments with the AEPFM.

Among the acrylamide-based AEPFMs, AC-AA and AC-MAA, the methyl-free AC-AA exhibits higher ion conductivity. Similarly, within the acrylate-based AEPFMs, AC-AE and AC-MAE, the methyl-free AC-AE shows higher ion conductivity. The structures of AA and AE are simpler, and without methyl groups, their molecular sizes are smaller, leaving double bonds unaffected. Conversely, MAA and MAE contain methyl groups, resulting in relatively larger molecular sizes which influence the double bonds. Therefore, the monomers are more flexible and reactive without methyl groups and more rigid and stable with them. All anion-conducting electrolyte monomers have hydrogen bond acceptors, where the carbonyl oxygen (C=O) serves as a hydrogen bond acceptor to retain moisture. However, the presence of methyl groups may limit hydrogen bond formation and restrict water molecule movement, thereby reducing ion conductivity. Additionally, acrylamide-based AEPFMs demonstrated higher ion conductivity than acrylate-based AEPFMs. This difference arises because AA and MAA both have an N-H hydrogen bond donor and a C=O hydrogen bond acceptor, whereas AE and MAE lack a hydrogen bond donor, possessing only a hydrogen bond acceptor.

**Figure 4:**
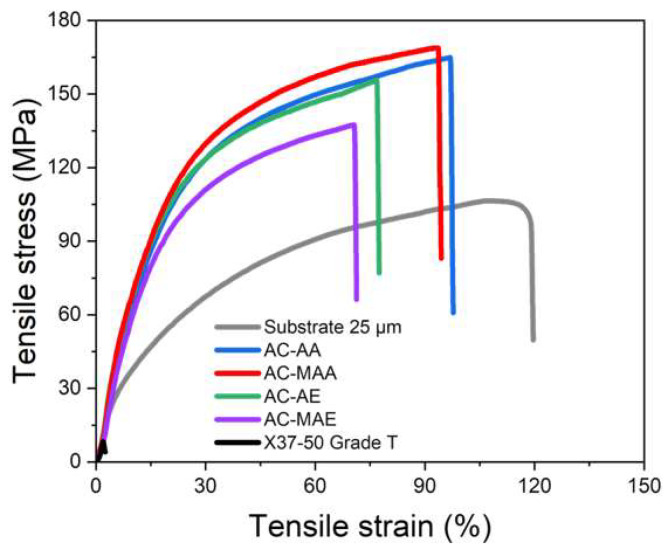
Stress–strain curves of the PE substrate, AEPFMs, and commercial AEM.

The mechanical properties of the porous PE substrate, AEPFMs, and commercially available Sustainion^®^ AEM (X37-50 Grade T, North Kingstown, RI, USA) are shown in Figure 4 and Table 3. The PE substrate displays a tensile strength of 106.8 MPa and an elongation of 119.7%. The AEPFMs exhibit even higher tensile strength than the PE substrate, with the combined properties of the substrate and the ionomer (an ACP) contributing to this enhanced strength. Both acrylamide-based and acrylate-based AEPFMs show greater elongation when using electrolyte monomers without methyl groups as opposed to those containing methyl groups. This aligns with the pattern in Table 3, where monomers with methyl groups display higher structural complexity than those without. The tensile strength and elongation of the AEPFMs demonstrate exceptional mechanical properties, approximately 16 times and 28 times greater than the tensile strength (8.30 MPa) and elongation (2.52%) of the commercially available AEM. Notably, these improvements occur even though the AEPFMs with 28–30 µm are nearly twice as thin as the 50 µm X37-50 Grade T membrane.

**Figure 5:**
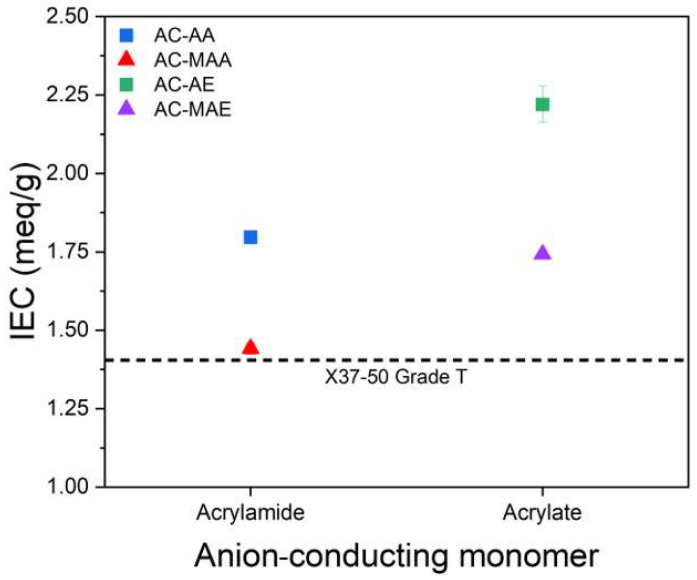
Variation in the IEC of AEPFMs (square and triangle symbols) and the commercial AEM (dotted line).

The IEC reflects an AEM’s ability to exchange anions and is determined by the quantity of cationic functional groups present. The ion exchange capacity results based on the structure of the anion-conducting monomer are shown in Figure 5 and Table 4. Theoretical ion exchange capacity was calculated from the repeating unit structure used during the synthesis of the monomer and cross-linker. Both the acrylamide-based and acrylate-based AEPFMs showed higher ion exchange capacities when anion-conducting monomers without methyl groups were used compared to those with methyl groups. Additionally, the acrylate-based AEPFM exhibited greater ion exchange capacity than the acrylamide-based variant, and this trend was consistent with theoretical calculations. Table 4 shows that the theoretical ion exchange capacity decreases when a methyl group is present due to the increased mass of the ACP, although the reduction is minor given the addition of just one methyl group per repeating unit. However, in experimental measurements, the ion exchange capacities for AC-MAA and AC-MAE with methyl-containing electrolyte monomers were significantly lower. This suggests that these more hydrophobic, methyl-containing monomers have reduced cross-linking reactivity with the cross-linking agent, considering the 16:1 molar synthesis ratio of electrolyte monomer to cross-linking agent.

**Figure 6:**
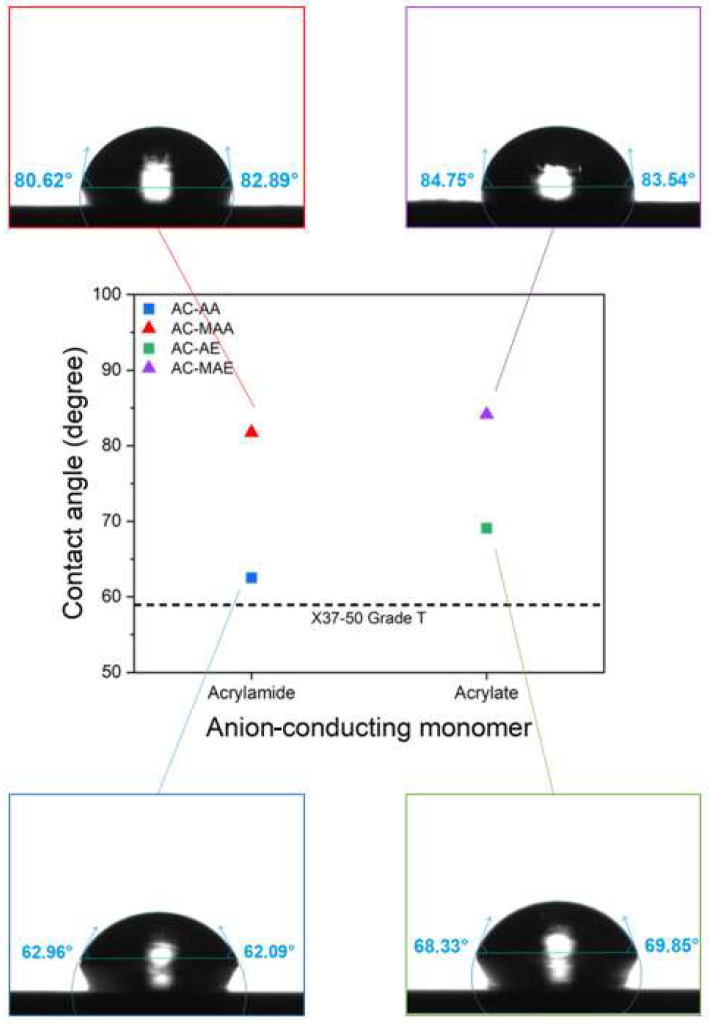
Contact angle of the AEPFMs (square and triangle symbols) and the commercial AEM (dotted line).

The contact angle was measured to directly confirm the degree of hydrophobicity in AEMs using electrolyte monomers with and without methyl groups. Figure 6 presents the contact angle results according to the structure of the anion-conducting monomers. Between AC-AA and AC-MAA, the surface of AC-MAA, which contains a methyl group, shows a higher contact angle. Similarly, for AC-AE and AC-MAE, the methyl-containing AC-MAE exhibits a higher contact angle. A higher contact angle indicates a more hydrophobic surface, making it more challenging for water to be absorbed into the membrane. Consequently, the increased hydrophobicity of AC-MAA and AC-MAE due to the presence of methyl groups reduces water absorption and hinders ion movement within the membrane. This aligns with the observed trend of lower ion conductivity and IEC in AC-MAA and AC-MAE compared to AC-AA and AC-AE, as shown in Figure 3 and Figure 5, respectively. In the case of X37-50 Grade T, the contact angle is 58.94°, which is lower than that of AEPFM, indicating greater hydrophilicity and thus higher ion conductivity (~64 mS/cm or ~0.08 Ω·cm^2^), consistent with the observed trend.

**Figure 7:**
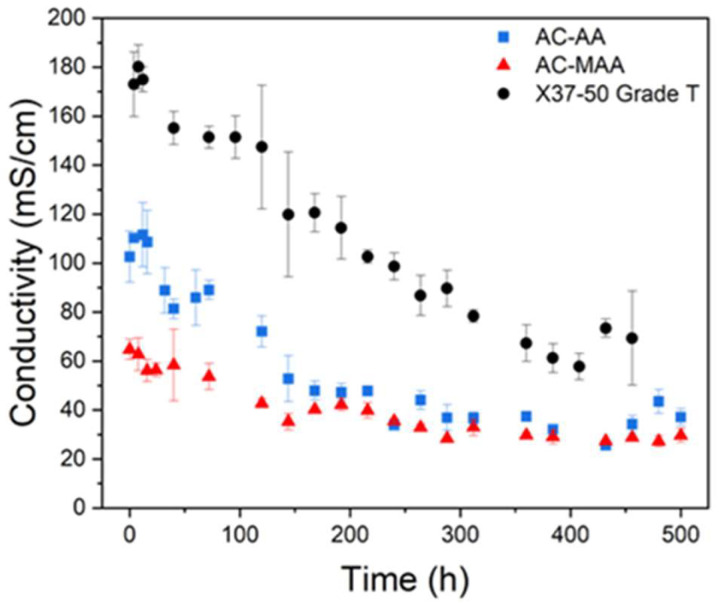
Variation in the ionic conductivity of AC-AA, AC-MAA, and the commercial AEM in 4 M KOH and 60 °C.

Since AEMWE operates in a highly alkaline solution, such as 1 M KOH, over extended periods, the alkaline stability of AEPFMs is crucial. An alkaline stability test was conducted to assess how well ionic conductivity is maintained when an AEPFM is exposed to an alkaline environment long-term. The results of the stability evaluation for the high-conductivity, acrylamide-based AEPFM are presented in Figure 7. AC-AA shows a 63.8% reduction in ionic conductivity, while AC-MAA, containing a methyl group, shows a 54.2% reduction. The improvement in alkaline stability for AC-MAA is attributed to the methyl group, an electron-donating alkyl group, which increases electron density and resistance to nucleophilic attack [42]. Given that X37-50 Grade T exhibited a 59.9% decrease in ionic conductivity, AC-MAA demonstrates superior alkaline stability.

**Figure 8:**
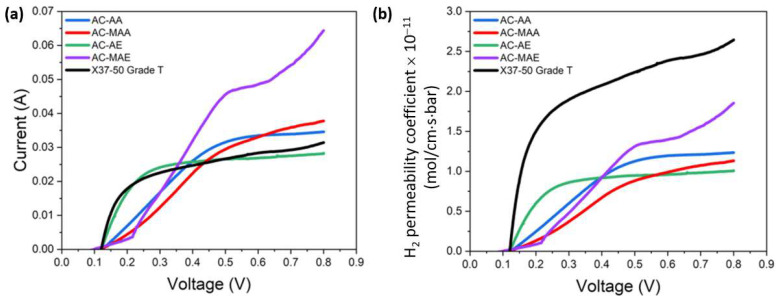
(**a**) Hydrogen crossover current and (**b**) hydrogen permeation coefficient curves as a function of applied voltage in an electrolyzer single cell.

In water electrolysis systems operated at elevated temperatures and under pressure, hydrogen crossover occurs when generated hydrogen permeates back through the AEM, compromising system stability and reducing efficiency. This phenomenon is mainly due to membrane defects, thinness, pressure difference, and concentration gradients. In particular, if the membrane lacks uniformity, crossover may occur more readily in specific areas. Consequently, hydrogen crossover indirectly indicates the membrane’s pressure resistance and uniformity. As shown in Figure 8, the hydrogen crossover current measured for the AEPFM at 0.8 V was generally higher than that of X37-50 Grade T (0.031 A). However, the hydrogen permeability coefficients of all the AEPFMs (0.6–1.7 × 10^−11^ mol/cm·s·bar) were lower than the 2.6 × 10^−11^ mol/cm·s·bar of the thicker X37-50 Grade T, confirming that the AEPFMs are properly manufactured and have higher pressure resistance than the commercial AEM. Generally, as membrane thickness increases, hydrogen permeability tends to decrease or stabilize. In this experiment, however, an opposite result was observed. The increased thickness of X37-50 Grade T, which is greater than that of the AEPFMs, had a stronger impact on crossover than it did on reducing permeability. In addition, no effect of the electrolyte monomer with methyl groups on hydrogen permeation was found.

**Figure 9:**
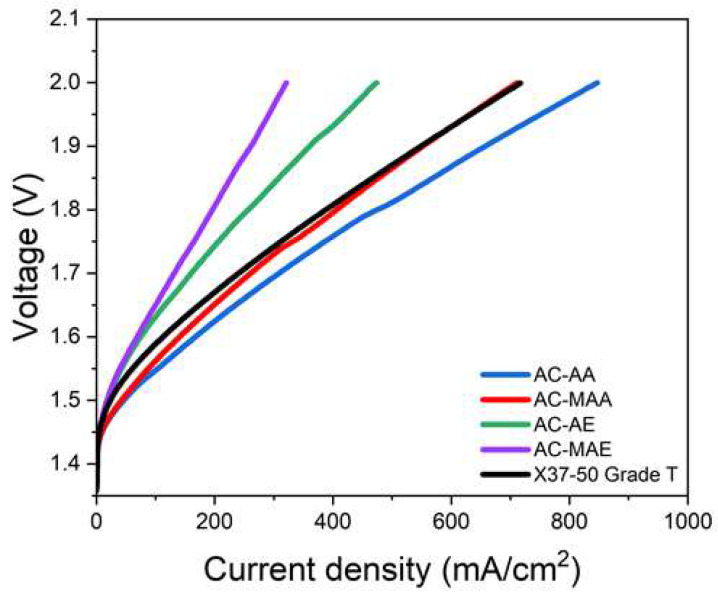
Performance curves of AEMWE using the AEPFMs and the commercial AEM.

The electrolysis performance results based on anion-conducting monomer structure are shown in Figure 9. At 1.8 V, current density followed the order of AC-AA > AC-MAA > AC-AE > AC-MAE. This indicates that both acrylamide-based and acrylate-based AEPFMs perform better without a methyl group in their structure, with the acrylamide-based AEPFM outperforming the acrylate-based variant. These findings align with the trend in ion conductivity. For X37-50 Grade T, a current density of 389 mA/cm^2^ was recorded at 1.8 V, while the acrylamide-based AEPFM achieved an even higher current density. It is believed that the superior performance of the AEPFM without a methyl group in an electrolysis environment is not solely due to the membrane’s inherent ion conductivity but also because the KOH solution at the anode effectively penetrates the pore-filled ACP layer on the AEPFM surface, establishing a rapid ion-exchange equilibrium.

**Figure 10:**
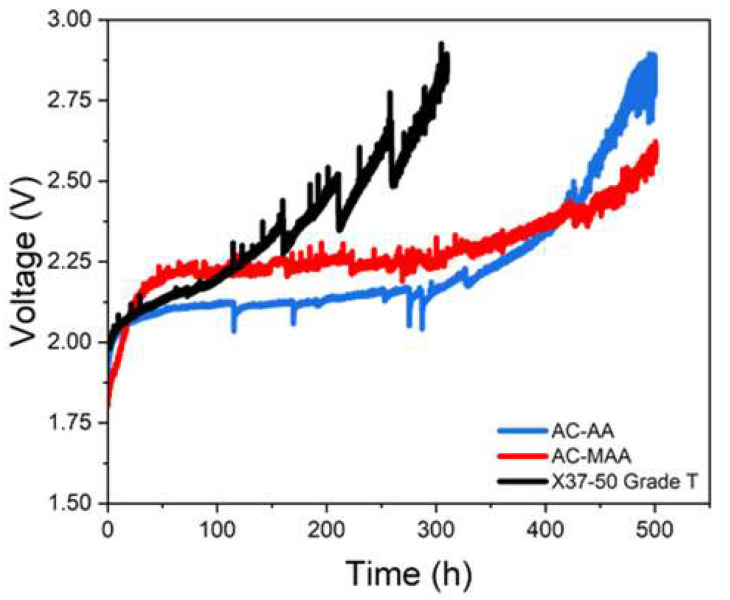
Variation in voltage at 0.5 A/cm^2^ as a function of AEMWE operation time for AC-AA, AC-MAA, and the commercial AEM.

The durability evaluation results based on the anion-conducting monomer structure are shown in Figure 10. In this evaluation, AC-AA demonstrates an increase rate of 1150 μV/h, while AC-MAA exhibits a lower increase rate of 746.6 μV/h, indicating better durability for AC-MAA. This enhanced durability is likely due to the improved alkaline stability provided by the methyl group. By comparison, X37-50 Grade T shows a decrease rate of 3116 μV/h, confirming that the AEPFM achieves superior durability.

While ACP-like methyl groups, which increase ACP hydrophobicity, can enhance durability by stabilizing AEMs in high-alkaline conditions, they can also impair performance. Nonetheless, AC-AA and AC-MAA exhibit both higher durability and performance compared to the commercial AEM. This suggests that when the ACP properties within the pores are well-controlled, AEMs with pore-filling membrane technology can offer greater performance and durability in electrolysis environments than homogeneous membranes.

## 4. Conclusions

This study investigated the impact of polymer hydrophobicity on AEPFMs and their potential for enhanced durability and performance in water electrolysis. By using two types of electrolytes, one with and one without a hydrophobic methyl group, conductivity, strength, and stability of the AEPFMs were evaluated. Methylated polymers, which are more hydrophobic, showed a higher elastic modulus. FT-IR spectroscopy confirmed successful polymerization and pore-filling, ensuring structural integrity. A molar ratio of 16:1 for anion-conducting to cross-linking monomers provided an ideal balance of ionic conductivity and mechanical strength, marking it as optimal for AEPFMs. Methyl-free monomers, such as acrylamide-based AC-AA and acrylate-based AC-AE, showed higher ion conductivity than their methylated counterparts (AC-MAA and AC-MAE). Methyl groups hinder water absorption, reducing ion conductivity and limiting water molecule mobility within the membrane. Both the acrylamide- and acrylate-based AEPFMs had significantly higher tensile strength and elongation than the commercial AEMs, especially without methyl groups. Even with reduced thickness, the AEPFMs demonstrated better durability and stability under high-alkaline conditions. In highly alkaline environments, methyl groups enhance stability, with the methylated AEPFMs (e.g., AC-MAA) showing reduced conductivity degradation over time. The AEPFMs also presented lower hydrogen permeability than the thicker commercial membranes, affirming their superior resistance to pressure and hydrogen crossover. Methyl-free AEPFMs performed better in water electrolysis, with the highest current density in AC-AA, suggesting that these membranes support rapid ion exchange. The AC-MAA variant, however, displayed better durability due to the stability provided by methyl groups. Overall, AEPFMs with controlled hydrophobicity and optimal monomer ratios outperformed the commercial AEM in both durability and electrolysis efficiency, indicating strong potential for long-term, high-performance applications.

## Figures and Tables

**Table 1:** Chemical structure, 3D chemical structure, and chemical name of electrolyte, cross-linker, and photo-initiator.

Type	Chemical Structure	3D Chemical Structure ^1^	Chemical Name (Code)
Electrolyte	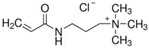	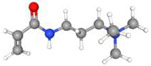	(3-acrylamidopropyl) trimethylammonium chloride (AA)
	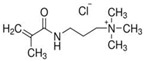	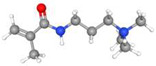	[3-(methacryloylamino)propyl] trimethylammonium chloride (MAA)
	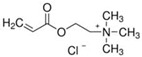	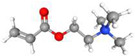	[2-(acryloyloxy)ethyl] trimethylammonium chloride (AE)
	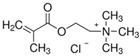	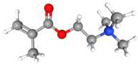	[2-(methacryloyloxy)ethyl] trimethylammonium chloride (MAE)
Cross-linker	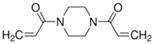	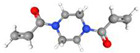	1,4-bis(acryloyl)piperazine (AC)
Photo-initiator	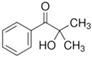	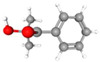	2-hydroxy-2-methylpropiophenone (-)

^1^ Grey, red, blue, and white atoms represent carbon, oxygen, nitrogen, and hydrogen, respectively.

**Table 2:** Key components and conditions of performance and durability measurement in an electrolyzer single cell.

Item	Materials and/or Conditions
Anode electrocatalyst	IrO_2_ (43396, Alfa Aesar)/1.0 mg_IrO2_/cm^2^
Cathode electrocatalyst	Pt/C (TEC10F50E, Tanaka)/0.4 mg_Pt_/cm^2^
Anode porous transport layer (PTL)	Titanium-PTL (2GDL09N-025, BEKAERT)
Cathode gas diffusion layer (GDL)	Carbon-GDL (20-A3, JNTG)
Electrode coating	Electrode-coated Ti-PTL and gas diffusion electrode
Electrode active area	9 cm^2^
Feed solution	1.0 M KOH
Feed solution flow rate	30 mL/min
Feed solution and cell temperature	60 °C
Durability test	Constant current mode at 0.5 A/cm^2^

**Table 3:** Tensile strength and elongation at break for the PE substrate, AEPFMs, and commercial AEM.

Sample		Tensile Strength (MPa)	Elongation at Break (%)
PE substrate		106.8	119.7
AEPFM	AC-AA	164.8	97.8
	AC-MAA	168.8	94.4
	AC-AE	155.8	77.5
	AC-MAE	137.4	71.3
X37-50 Grade T		8.3	2.5

**Table 4:** Theoretical and experimental ion exchange capacities of AEPFMs and the commercial AEM.

Sample		Ion Exchange Capacity (meq/g of Dry Membrane)	
		Theoretical	Experimental
AEPFM	AC-AA	2.45	1.80 ± 0.02
	AC-MAA	2.37	1.44 ± 0.02
	AC-AE	2.53	2.22 ± 0.06
	AC-MAE	2.45	1.74 ± 0.01
X37-50 Grade T		-	1.40 ± 0.03

## Data Availability

The data that support the findings of this study are available from the corresponding author upon reasonable request.
